# The decision-making process for the fate of frozen embryos by Japanese infertile women: a qualitative study

**DOI:** 10.1186/1472-6939-13-9

**Published:** 2012-05-20

**Authors:** Shizuko Takahashi, Misao Fujita, Akihisa Fujimoto, Toshihiro Fujiwara, Tetsu Yano, Osamu Tsutsumi, Yuji Taketani, Akira Akabayashi

**Affiliations:** 1 The Department of Obstetrics and Gynecology, The University of Tokyo School of Medicine, 7-3-1 Bunkyo-ku, Tokyo, Hongo 113-0033, Japan; 2 The Department of Biomedical Ethics, The University of Tokyo, Graduate School of Medicine, 3-1 Bunkyo-ku, Tokyo, Hongo 113-0033, Japan; 3 Reproduction Center, International University of Health and Welfare, Graduate School, 1-3-3, Minami Aoyama, Tokyo, Minato 107-0062, Japan

## Abstract

**Background:**

Previous studies have found that the decision-making process for stored unused frozen embryos involves much emotional burden influenced by socio-cultural factors. This study aims to ascertain how Japanese patients make a decision on the fate of their frozen embryos: whether to continue storage discard or donate to research.

**Methods:**

Ten Japanese women who continued storage, 5 who discarded and 16 who donated to research were recruited from our infertility clinic. Tape-recorded interviews were transcribed and analyzed for emergent themes.

**Results:**

A model of patients’ decision-making processes for the fate of frozen embryos was developed, with a common emergent theme, “coming to terms with infertility” resulting in either acceptance or postponing acceptance of their infertility. The model consisted of 5 steps: 1) the embryo-transfer moratorium was sustained, 2) the “Mottainai”- embryo and having another child were considered; 3) cost reasonability was taken into account; 4) partner’s opinion was confirmed to finally decide whether to continue or discontinue storage. Those discontinuing, then contemplated 5): the effect of donation. Great emotional conflict was expressed in the theme, steps 2, 4, and 5.

**Conclusions:**

Patients’ 5 step decision-making process for the fate of frozen embryos was profoundly affected by various Japanese cultural values and moral standards. At the end of their decision, patients used culturally inherent values and standards to come to terms with their infertility. While there is much philosophical discussion on the moral status of the embryo worldwide, this study, with actual views of patients who own them, will make a significant contribution to empirical ethics from the practical viewpoint.

## Background

The development of embryo cryopreservation and frozen embryo transfer has been useful for allowing future pregnancy attempts and reducing the risk of multiple pregnancies by limiting the number of embryos transferred [1]. However, as a result, an enormous supply of embryos in storage has been reported worldwide [2-4]. In Japan, an estimated 61,000 embryos remain cryopreserved in storage nationwide, 15 % stored without definite plans for usage [5]. Most patients store their unused embryos; however, after a certain period, the patient needs to make a decision on the fate of frozen embryos.

The storage period varies amongst countries and institutions. The Japanese Society of Obstetrics and Gynecology (JSOG) regulates the storage limit “until the end of the woman’s reproductive life (without a definite age or clear definition)” [6] and most institutions in Japan have a set storage period due to maintenance and storage cost. At the time of cryopreservation, patients are obliged to give consent to embryo disposal when they cannot be contacted after a set period of storage and in the case of divorce or a death of a partner. At the end of the storage period, patients must choose between three options: continue storage by paying an additional cost, discard, or donate to research. JSOG prohibits embryo donation to other infertile persons or couples since it causes confusion in parent–child relationship and the child’s welfare needs to be most prioritized [6].

The emotional burden in making this decision has been reported previously by several studies [7-14], with evidence that the disposition preferences changed over time [7,9,13] and those more worried about their embryos had longer storage time or wanted to freeze them indefinitely [8,11,15]. Factors influencing the decision have been found. These include: conceptualization of the embryo [9,10,12,14,16-19]; confidence (trust) in medical science [14,16,20]; and the lack of acceptable options [14]. In addition, gender difference [8,21], and disagreement between couples [11] concerning these factors has been reported. However, the majority of these studies focused only on the decision to dispose, not having both options of continuing and discontinuing storage; some of such studies were in recognition of the laws limiting storage time [8-14]. Furthermore, most of these studies reported influential factors but the actual decision making process remains unclear.

Only a few conceptual models, helpful in understanding the actual process, have been reported, using a qualitative approach [14,18,19]. Provoost et al. showed the embryo disposition decision in 2 stages: first, considering donation to others for reproductive purposes; and, second, considering donation for science [14]. Nachtigall et al. illustrated the 3 questions to reach a decision and conflict with partners was observed in relation to the decision about whether the embryos were to be used for conception [19]. However, these studies included patients who have yet made their decision, so these models may not reflect the actual process of those making such decisions since many studies show that patients often change their decision at a later stage [7,9,13]. In addition, much of the data obtained were from couples, and some may have felt constrained to speak of their real feelings in the presence of their partner.

Socio-cultural and demographic factors such as religion, treatment period [8,22], ethnicity [22,23], income, marital status, and education have been reported as influential in terms of donation to research [22]. Choudhary et al. found that ethnic minorities were less willing to donate their embryos to research [23] while, Jain and Missmer found that compared to Caucasians, and in particular Protestants, Asians and those practicing other religions overwhelmingly approved the use of embryos for stem cell research [22]. In Japan, despite arguments that religious influence is weak and less burden is felt compared to western societies, it has been reported that cultural values towards the embryo, such as Motherhood ethics, causes emotional burden during in vitro fertilization (IVF) treatment [24]. Therefore, it is unclear if the results of the past qualitative studies are applicable for Japanese patients having continued storage as an option.

It has been criticized in Japan that patients can hardly bring themselves to express their feelings about embryos in the clinical settings [24]. Therefore, there is a need for effective counselling and informed consent methods developed for patients who have difficulties making decisions about the fate of their frozen embryos. Hence, an understanding of the psychological processes of patients with different socio-cultural backgrounds that influence the decision is required. We therefore conducted a qualitative interview study of Japanese infertile women who have just made a decision concerning their embryos. This study aims to construct a conceptual decision-making model and to identify the socio-cultural factors that influence these decisions.

## Methods

### Design

The qualitative research strategy, specifically a semi-structured in-depth interview, was used to explore how patients decide the fate of their cryopreserved embryos. Approval of this study was obtained from the Research Ethics Committee of the University of Tokyo, Graduate School of Medicine.

### Participants

To be included in this study, participants were required to have received IVF treatment at the University of Tokyo Hospital, IVF Center and to have or have had embryos in storage. Upon cryopreservation, a contract is made with the patient at our IVF Center to store the surplus embryos for a period of three years. Approximately one month prior to the expiration date, letters of notice are sent so that the patient can choose from three options: continue storage for another 3 years by paying 70,000 yen (approximately 833 U.S. dollars as of March, 2012); discard; or donate to research at our institute. If there was no response or in the case of returned mail, the embryos were discarded, according to initial informed consent. Our recruiting letters for interview were sent to those who responded to the letters of notice. Unlike previous studies focusing on disposition, we also interviewed patients who decided to continue. Since patients’ attitudes towards cryopreserved embryos are known to change over time, we specifically interviewed participants who had just recently made a decision regarding the fate of their embryos. Sampling was completed when we reached “theoretical saturation,” and no new or relevant data seem to emerge regarding a category [25]. Therefore, we completed our sampling after having conducted 31 interviews (10 who continued storage, 16 who donated to research and 5 who discarded). Out of the 31 interviewees, 28 had children and 3 were without children. The demographic information of the participants is as shown in Table 1.

**Table 1:** Demographic composition of interviewed participants

Number of patients interviewed		31
	Continue Storage	10(32.3 %)
	Donate to Research	16(51.6 %)
	Discard	5(16.1 %)
Age, years (range)		38.8 (31 ~ 45)
	Continue Storage	38.7 (31 ~ 44)
	Donate to Research	38.8 (31 ~ 43)
	Discard	41.2 (38 ~ 45)
Number of stored embryos		
	1-5	23
	6-10	5
	11 or greater	3
Pregnancy and Delivery		
	IVF Pregnancy	28
	Natural Pregnancy Only	2
	No Pregnancy	1
	IVF Child	25
	Naturally Conceived Child post IVF	5
	No Child	3
Education		
	High School graduate	4(12.9 %)
	Community College graduate	12(41.9 %)
	University graduate	13(41.9 %)
	Graduate School graduate	1(3.2 %)
Employment		
	Employed	13(41.9 %)
	Unemployed	18(58.1 %)
Reason for infertility (some chose multiple reasons)		
	Ovarian factor	4
	Tubal factor	5
	Uterine factor	3
	Endometriosis	4
	Male factor	8
	Unexplained	11
Religion		
	Buddhist	10(32.3 %)
	Tenri-Kyo (monotheistic religion)	1(3.2 %)
	No Affiliation	20(64.5 %)

### Data collection

Interviews took place between July 2007 and February 2009. All the interviews were conducted by the first author (ST), a certified Obstetrician and Gynaecologist and not directly involved in their treatment. For those who had agreed to be interviewed, an explanation of the objectives and procedures of the research was given, and informed consent was obtained. Semi-structured interviews were carried out in a private room located within the hospital unless otherwise suggested by the participant. Interviews lasted 50 minutes on average and ranged in duration from 32 to 118 minutes.

An interview guide, developed from pilot interviews with patients who had yet to make their decision and relevant literature, was used to ensure the objectives of the research were met. The interview guide included questions such as “How did you make your final decision?”, “How do you feel about the options you did not choose?”, “Did you think of the possibility of alternative choices?”, “How does your partner feel about the embryo?”, and “Who else influenced your choice?” We began each interview by asking participants to answer open-ended questions about how they made their decision and what it was like having embryos cryopreserved. Over the course of time and as the analysis of the interviews progressed, the interview questions were altered using constant comparative analysis.

All interviews were audio taped and transcribed verbatim. To increase credibility of the themes, the results were discussed at conferences and seminars with physicians, embryologists, nurses, and counsellors involved in IVF. In addition the results were presented to IVF patients at other hospitals who had just made the same decision.

### Analysis

All transcripts were analyzed using a qualitative data analysis software program, ATLAS.ti.5.2 (Scientific Software Development GmbH, Berlin). From the first interview, we followed a “grounded theory” approach and the transcripts were coded using open coding (identifying concepts in data), axial coding (relating categories to their subcategories), and selective coding (integrating and refining the theory) [25]. Throughout the coding process as the interviews progressed, using constant comparative analysis, the investigators reviewed the codes to determine how certain codes form a certain category. These categories were then organized to describe the central phenomenon.

## Results

**Figure 1:**
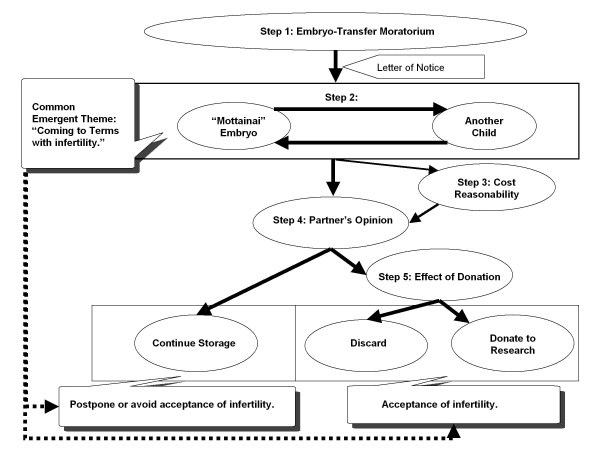
The decision-making model for the fate of frozen embryos.

Based on grounded theory, a model of the decision-making processes for the fate of frozen embryos was developed as shown in Figure 1. For all patients, the process involved a common emergent theme: “coming to terms with infertility,” associated especially with how the embryo was considered: “Mottainai” {“Mottainai” is a Japanese word with three meanings: 1) impious; sacrilegious, 2) more than one deserves; be unworthy of, and 3) wasteful; wasting [26]. It is an ethical value taught in school, in homes from early childhood and its importance emphasized even on national TV commercials [27]. For example, a young child is scolded “Mottainai!” for leaving rice in the bowl. This is not just because it is being wasteful but since it is immoral, shameful and arrogant to leave rice that is bestowed upon with farmer’s hard work, and considering those who are starving, requires appreciation.}. Based on this theme, there was a 4 step decision-making process for those who continued storage and one additional step for those who discarded or donated their embryos to research. The steps were as follows: 1) the embryo-transfer moratorium was sustained; 2) the “Mottainai”-embryo and another child were considered; 3) cost reasonability was taken into account; and 4) partner’s opinion was confirmed to finally decide whether to continue or discontinued storage. For those who decided to discontinue, 5) effect of donation was then contemplated to decide whether to discard or donate to research. Although all steps involved emotional stress, greater psychological strain and conflict was especially expressed in steps 2, 4 and 5.

### Common emergent theme: “coming to terms with infertility”

All participants, whether or not they had children with treatment, described a consistent difficulty throughout this entire decision-making process, with confronting their infertility due to the embryo having “Mottainai” value, also a critical factor in Step 2. This was since most believe that these embryos were their only means of conception, such that discontinuing storage was directly related to “the loss of lifetime fertility.” The embryos represented a belief that the patient was nearly a “normal” woman capable of conceiving when desired. Therefore, prior to the letter of notice, with or without having a child, infertility seems like a distant problem. However, a participant who had successfully had a child said:

"Receiving that letter was like a bell telling my time to conceive is over, lost in the battle of conception. I know I shouldn’t think this way, but having those embryos is like having insurance to my fertility. Normal women do not have to opt to terminate their conception and my infertility stigma revisited."

So, sense of being “infertile” remained with them despite having a child after IVF.

Many participants described relief once the decision to continue cryopreservation was made, since they were able to postpone or avoid infertility acceptance for another 3 years. Some valued the “Mottainai”-embryos so much, even after a having a child that they wished to store indefinitely, if allowed:

"The decision is made now and I’m at ease. It’s not like there is ‘no hope’, I’m not completely infertile…. The next decision (discontinuing storage) is so horrifying that I wish to continue storage until I die."

There was also an inner shame associated with disposal of embryos because many infertile couples, at one time considered their peers, are not so privileged to even make that decision:

"Discarding is too “Mottainai” considering waste of all the hardships attaining it and inconsiderate to those patients who could not even reach that point of having embryos."

Therefore, the embryo was “Mottainai” since it is more than one deserves as well as wasteful of “what alleviates feeling stigmatized as an infertile woman.” Furthermore, many participants mentioned their male partner not feeling this conflicted regardless of which partner was responsible for the infertility issue, which lead to their inability to mutually understand the psychological burden.

Those who discontinued accepted their infertility by seeking ways to overcome their emotional burden. Participants adopted several coping styles, including sublimation; letting go; and “Kuyo” (a culturally specific Buddhist ceremony practiced by both religious and non-religious people). Donating to research became sublimation of guilt for not utilizing their “Mottainai” embryo. By helping IVF children and other infertile couples, as they once were, was “an ideal end to their infertility story.” As a participant expressed her relief:

"I don’t know what I would have done if the option to donate was unavailable. Such a perfect reason, now I can be glad that I had the IVF experience."

For many discarding stored embryos equated to liberation from the entire infertility experience. Participants expressed discarding as “letting go”, so to move forward to a world unrelated to infertility by detaching themselves from their “Mottainai”-embryo.

Others had alternate ways as “Kuyo,” a memorial service for the dead or things no longer used, and as a method to pay tribute for their effort during their activity [28]. As in the “Mottainai”-embryo in Step 2, they were “life, child, and a part of themselves,” “what came to them linked by *go-en* (fate and fortune),” and “a symbol for hardships of IVF treatment.” So, “Kuyo” was utilized not only to overcome the emotional burden of discarding the embryo, but also to accept infertility:

"I feel the need for a ‘Kuyo’ for those embryos of course, but rather for myself to console the burden of having to receive infertility treatment and having to make this decision."

### Step 1: Embryo-transfer moratorium sustained

Here, after a successful pregnancy, or while ceasing treatment, child bearing and rearing, work or daily life become so important to the patient that embryos in storage become of secondary importance, inhibiting embryo transfer to the uterus. Eagerness towards pregnancy and expectations towards the embryo are decreased, compared with behavior during treatment. As one claimed, “Child rearing was so unexpectedly hard” that they “almost forgot the happiness of having the embryo or pregnancy using it.” However, participants kept the embryos in storage since they afford, “a chance of another pregnancy attained while undergoing treatment hardships.” Having the embryos was often expressed as “insurance” for their fertility, leaving the embryo to be transferred to the uterus in moratorium. Participants prolonged this “embryo-transfer moratorium” state, and remained indecisive about embryo utilization prior to the letter of notice. Simultaneously, during this state, the patients felt two profound pressures to make a decision about their embryos. First, a time limit: storage time and age limitation for child bearing and rearing, which may be set by themselves, family or physicians. Second, the perceived pressure of having another child: requests of a sibling from their own child, and culturally specific social pressures of an only child. The arriving letter of notice initiates the decision-making process, or acts to break the ice associated with this indecisive state:

"I went as far as IVF to make the embryo. I knew my responsibilities to think hard. Time just passed, friends getting pregnant again, made think more. Finally, that letter arrived; I HAD to think more intently."

### Step 2: “Mottainai”-embryo and having another child are considered

After the letter of notice arrived, all patients began considering the significance of the embryo to themselves, the “Mottainai”-embryo, as well as having another child by embryo-transfer in the future. Some considered the “Mottainai”-embryo foremost, while others started from thinking about having another child. The word, “Mottainai,” was used without the researchers introducing the word.

#### “Mottainai”-embryo

In addition to the embryo meaning “lifetime fertility” to the participants discussed earlier, the embryo was “Mottainai,” since they were “life, child, and a part of themselves,” “what came to them linked by *go-en* (fate and fortune),” and “a symbol for hardships of IVF treatment.” The psychological burden involved in discontinuing or continuing storage, the embryo’s relationship to the existing child, and the cryopreservation technique were all evaluated to determine the worthiness of transferring or storing the embryos. Some claimed a lack of information regarding their embryos, particularly concerning the quality, quantity and the probability of implantation, made this evaluation difficult. Five of the participants actually visited our clinic for this information.

The value of the embryo was affirmed and the desire expressed to store it with the hope of possible future transfer if the participant felt that the burden of disposal could not be withstood; the embryo (as a sibling of the existing child) would have high pregnancy potential; and/or gratitude toward, and understanding of, the cryopreservation technique:

"They were created and disposed from the parental ego… It is too “Mottainai” that not returning it to where it belongs- the womb- will leave me as guilty if I had an abortion."

At this point, the participants then considered having another child in the future.

In contrast, if patients felt that having the embryo remain in storage was a burden (from fear the existing child may discover their mode of conception and obligations remaining towards the embryo), and they felt that the embryo as “unnatural” becoming a sibling of the existing child, the embryos were considered unworthy of transfer:

"Freezing, thawing and returning it: the entire process so abnormal and unnatural. Though they are mine and are “Mottainai”, it is better if the embryos are made more naturally…. not this way."

Participants also rationalized that the embryos with high pregnancy potential had been somehow consumed and used up by the existing child since embryo transfers are performed using the better ones. However, some patients in the study displayed strong ambivalence because of a sense of guilt assumed because the embryo is “Mottainai.” This resulted in then contemplating the possibility of having another child in the future.

#### Another child

When considering the use of “Mottainai”-embryos, participants appeared to reconfirm their family planning, re-evaluate the IVF experience from their initial decision to receive IVF up to child rearing, and review the feelings towards embryo-transfer as a mode of conception. Many solicited advice from physicians and other family members to confirm their physical capability and availability of child support for another treatment.

Having another child in the future was believed to be plausible if their family was felt to be incomplete and if their IVF experience was perceived positive, alongside ongoing support from other family members. These participants felt that embryo-transfer was their only means of conception.

Conversely, another child was undesired if their family planning was felt to be complete, their IVF experience was perceived negatively, and if there was a lack of support from family members or peers:

"Underneath, I have always perceived my child as different from ‘normal’ children, a result of IVF, and as that embryo. So, this time, I want to get pregnant, normally, and naturally."

These participants felt embryo-transfer unnecessary or not plausible because of a preceding natural pregnancy or being diagnosed incapable of having another child. Many wished for child support within the hospital and counselling (both not offered) in order to go through IVF treatment again.

For those wanting another child, if the “Mottainai”-embryos were worthy of transfer and storage, cost reasonability was then considered (step 3) with continuation of storage in mind. This decision was expressed as similar to “picking up a child left at the hospital”. On the contrary, for those not wanting another child, the “Mottainai”-embryos were deemed unworthy, participants directly went to step 4 with discontinuation in mind, and without much consideration to cost (step 3). These participants generally felt guilty for making surplus embryos initially, but often expressed this decision “as inevitable having received IVF.”

However, those not wanting another child, but who still considered the “Mottainai”-embryo worthy, were hesitant to make a decision and fluctuated between the two concerns: the “Mottainai”-embryo and another child:

"It is improbable that I would receive an embryo transfer to have another child, but at the same time, seeing my child now I feel resistant that a part of me, a child of mine, will be discarded by my word ‘discontinue.’ I feel as though I will be in this muddle forever."

This involved such an emotional conflict that some hoped for alternative choices, such as creating a “storage cemetery,” or donation to other couples, which is prohibited in Japan [6]. These participants proceeded to step 3 without a definite decision.

### Step 3: Cost reasonability

With continuation in mind, or a hesitancy to decide, participants appeared to contemplate whether the storage cost of 70,000 yen was reasonable for “fertility insurance” or as just storage. Those who felt the cost reasonable- since it was “not a matter of money, as much as an investment for hope”- went to step 4, thinking continuation. On the other hand, participants who felt the cost unreasonable, since it was “like gambling considering such low successful outcomes of transfer,” went to step 4, thinking discontinuation. Those who wanted to continue but could not due to the cost, suggested alternative ways: a contingency fee (paying only when the transfer outcome is a successful pregnancy) or a fee covered by medical insurance. Both are not possible options in Japan.

### Step 4: Partner’s opinion confirmed

All participants indicated that once their decision was roughly made, their male partner’s opinion was then confirmed. The participants reported that their partners appeared to have undergone the same thought processes although with less psychological burden since they were less attached to the “Mottainai”-embryo. This difference, originating from the initial decision to undergo IVF, was explained as men not “having to experience pregnancy” or “having to observe the entire process of IVF.” Some even expressed marital distress due to this difference.

When in disagreement, discussions began concerning the decision to have another child, to go through treatment again or to discontinue storage, involving a great deal of emotional conflict and strain. Participants often felt the need for more time to decide with their male partner and expressed the wish that the letter had come earlier, though the letters typically arrive one month before storage expiration date.

Continuation was chosen if the patient persuaded the male partner that the emotional burden of discontinuing the cryopreservation of the embryo was unbearable, or if she is successfully persuaded her desires for another child on the condition that the partner offered more child support:

"I didn’t want another child for he was so uncooperative with this child. But he convinced me that he’d help and now I can be optimistic to have another."

Also, continuation was chosen if an agreement could not be reached. To postpone the decision:

"It isn’t just an embryo to me, so I couldn’t give it up. Finally, he gave in saying that he ‘cannot feel the same way as before’ about the next step. So no embryo-transfer, just continue storage."

This discrepancy towards continuation appeared to be related to “coming to terms with infertility” mentioned earlier. Therefore, after the decision to continue was made, participants reported feeling at ease, at least for the time being, though some felt already scared of having to make a decision again 3 years later.

On the other hand, discontinuation was accepted reluctantly if the patient was persuaded the male partner that another child was not desired because of insufficient child support during treatment, or if the patient was persuaded to believe that embryo-transfer was unnecessary or natural pregnancy preferred:

"Really, though I am sorry towards those embryos, I still want to keep them. My husband is evermore against me going through the IVF process again, though."

Another patient in the study felt that her partner accepted discontinuation by understanding that the simultaneous burden of treatment and having another child would be heavier:

"In truth, he wants another, a boy. But it is hard for him to keep saying that, because he understands the burden of treatment is more upon the woman."

Some participants reported having difficulty in achieving an agreement, wishing that the option of embryo donation to other infertile couples were permitted, because of the guilt of non-use.

If in agreement, or if the decision was left up to the participant (since the burden of IVF was perceived to be more the woman’s), the decision to either continue or discontinue storage was made quickly.

### Step 5: Effect of donation contemplated

From this point onward, the patient and her partner were deciding together. Those who had decided to discontinue storage contemplated research donation since all participants were hesitant about discarding their “Mottainai”-embryo. As one participant expressed, in a raised voice:

"It is a symbol of my hardship: it is a crystallization of my blood, sweat, and tears! I cannot just throw it away!"

Patients and partners who had a negative image towards research (viewing it as an anatomical and invasive harm towards the “Mottainai”-embryo) described the research as untrustworthy, unclear and cumbersome with formalities. These couples chose to discard to avoid “another evil deed” or to “disrespect” the “Mottainai”-embryo:

"He saw it as making zombies or mummies, the dead forced to come to life. Better to discard, but just remember that our kids exist because of the embryos."

If the patient’s image of research was positive, she viewed it as making the best use of the “Mottainai”-embryo: as a way to restore the life of the “Mottainai”-embryo; and/or to identify IVF specific risk factors for the existing IVF child. Accordingly, donation was chosen. For these patients, donation was an expression of gratitude towards assisted reproductive technologies- their contribution to society- and some even felt salvaged from the guilt of not giving birth by donating to infertility research. One participant, who previously did not want to discontinue storage, expressed her relief:

"I can give up now. I didn’t discard, but dedicated my child and my IVF experience to the hospital."

Though varied in degree, all participants wished for a memoir of their embryo, such as a photo of the embryo or a copy of the research article published in which their embryos were used as material.

## Discussion

This is the first conceptual model from a non-western country, made on the decision making process for the fate of cryopreserved embryos with 3 options: to continue storage, to discard or to donate to research. In addition to the burden on those who disposed, as shown in the previous studies, those who chose to continue storage also felt great emotional burden. In this study, we found new factors associated with this emotional burden different from those previously elucidated. Emergent throughout the entire process, “coming to terms with infertility” was a central theme, which made the decision very difficult for many women. This was since the embryo had a unique value, “Mottainai”, determined to be of critical importance to Japanese infertile women deciding the fate of their cryopreserved embryos. Since these burdens becomes more severe by letter of notice, it is ethically important for medical staff to give informed consent as to how one’s feeling towards the embryos may change overtime at the time of its making to well prepare the patients. In the following paragraphs, we discuss the common emergent theme, the “Mottainai”-embryo, and the steps in the model involving emotional burden.

### Unique value: “Mottainai”-embryo

Previous studies have indicated the embryo’s moral status, its existence as a person (a child, early-life) or not, as being one of the major causes of emotional burden [10-12,14,15,18-20]. Our patients expressed this burden as “Mottainai,” a prevalent, culturally embedded moral standard in Japan. Although “Mottainai” has become recognized more widely recently in terms of environmental conservation introduced by the Nobel Peace Prize Ecologist, Wangari Maathai [29], the word is inherent in Japan; starting from the 11^th^ century [30]. “Mottai” is a Buddhist term that refers to the intrinsic dignity or sacredness of a material entity. “Nai” is negation [30]. Therefore, “Mottainai” is an expression of sadness and guilt over the disrespectful and wasteful treatment of valuable entities. Hesitance and guilt regarding disposal of embryos and/or resolution of decision-making thereof, were expressed as “Mottainai” by all the participants, and was linked to the burden of coming to terms with infertility.

The meaning of “wastefulness” in “Mottainai” overlaps with the West, as getting “some use out of them” when donation to research is selected [12]. But our patients by using this word, not only expressed wastefulness but shame. About half the participants felt shame toward other couples without embryos, expressing shock at wasting something for which they are very eager, making the embryo more than one deserves. No such report has been found. So, the “Mottainai” embryos may include moral values unreported by previous studies. It is difficult to compare since we did not interview patients from other cultures to show a clear distinction. It would be interesting to further cross-examine this with moral standards of other cultures.

### Step 2: Burden of considering continuing storage and having another child

In step 2, having another child in the future and the “Mottainai”-embryo were weighed in an attempt to complement each other, potentially causing much emotional strain for those who do not necessary want another child but could not decide to dispose of their embryo. In line with the previous studies [11,16]: the psychological burden involved in discontinuing storage; the embryo’s relationship to the existing child; the cryopreservation technique; family planning; and previous IVF experience up to child-rearing were factors that were evaluated. In addition, the psychological burden involved in continuing storage was a factor newly identified. Though those continuing storage were relieved for now, some remained intimidated by the deadline in another 3 years time. Those who visit the clinic for advice concerning these factors may carry this emotional strain. Therefore, it is critical for medical professionals to not just simply offer information requested, but also to identify the motives behind their patient’s visits with a sympathetic attitude. It might also be desirable for medical professionals to encourage and facilitate discussion amongst their partners and family, not least because the availability of child support and psychological support from family members during treatment were discovered to be very influential to the patient’s ultimate decision.

### Step 4: Burden caused by the male partner’s opinion

The importance of partner opinion confirmation was one of the most important decision making steps for women. Little discussion took place until then, so many couples appeared conflicted when presented with the topic. As with “coming to terms with infertility, and in line with a previous study [21] their male partners appeared to be less attached to the embryos from the time of its creation. As one would expect, our participants generally felt resentful that their male partner did not feel the same way at the same time and some claimed marital distress. Much past literature has emphasized the importance of couples seen together from the initial consultation, because infertility has been considered and treated as a couple’s issue [31,32]. More encouragement towards the couples to discuss the fate of their embryos, from the time of its creation may help prevent these conflicts. Male partners becoming more aware their partners are coping with “coming to terms with infertility” by her selves in addition may alleviate much burden. However, in this study, the actual decision-making process for the male partner remains unclear. Future investigation of the male partners’ rationale regarding the fate of stored embryos could further clarify the reason behind this observation.

### Step 5: Burden from cultural and ethical views on donation to research and discarding embryos

Emotional burden in Step 5 was felt mainly by those who discarded their embryos, since all who chose to discard did so while feeling conflicted about the decision. As with previous studies, reasons for not donating were lack of confidence in medical science [14,16]; the incomprehensibility [15] and inconvenience of the content of the research; and the embryo being recognized as a virtual sibling [9,12,14,18,24],]. As in Kato & Sleeboom-Faulkner’s study, we also found that the thought of dissecting an embryo or transforming it into another form was considered disrespectful by those opposed to donation [24]. However our participants’ decision seemed influenced by the cultural importance of maintaining bodily integrity, or valuing the body as whole on the death of a person [33] rather than from the cultural notion of motherhood having responsibility not to have their children cut up [24]. This may be due to our limitation of not interviewing their male partners and therefore we could not differentiate motherhood from fatherhood. Nonetheless, the results suggest that medical professionals should identify patients’ views about research, prior to offering research information, in order to obtain satisfactory consent.

Positively, those who donated felt alleviated from the guilt and shame of disposal of the “Mottainai” embryos. This effect has not been reported previously, however, a similar effect was seen in women opting to donate aborted fetuses for fetal tissue transplant, in order to alleviate the guilt of having an abortion [34]. Many of our participants were appreciative that this was an available option because of the alleviation of guilt. With more ethical discussions on the meaning of the embryo to the patient, alongside paying careful attention to research conduct, an increase in the number of institutions with this option might help to alleviate the guilt associated with disposal as well as helping many cope with the emotional strain.

### Common emergent theme: burden of “Coming to terms with infertility”

Despite many of our participants having had successful outcomes, most still viewed themselves as being infertile. Those who experience unwanted infertility are known to take on a “central identity of self as infertile”: viewing themselves as “not normal,” or “different from” fertile women of the same age [35,36]. How our participants saw themselves was quite similar to these findings. According to these studies, this identity continues up until menopause for some patients [36]. Further follow-up interviews would be required to determine what our participants, including those who seemed to have accepted their infertility, experience at menopause.

Previous studies have indicated that embryos are symbols of infertility, representing women’s negative infertility experience [18]. However, in our study, for many women it represented their fertility: having stored embryos was often expressed as having a chance at pregnancy and feeling close to being “normal.” One reason that this theme was brought forth may be attributed to the homogeneity of Japanese society, emphasizing the importance of “being the same as everyone else” and “being normal” [37,38]. The added pressures on Japanese infertile women not being able to have a child through natural conception is explained as a “social and physical stigma” by some Japanese researchers [39]. Indeed, birth as a result of IVF is still rare and is responsible for approximately 1.6 % of births in Japan as of 2004 [40]. However, the number of babies born by assisted reproductive technologies is not low compared to other countries [41]. Perhaps, “being the same as everyone else” may also be emphasized in other countries, so further cross-cultural studies are required to confirm whether this finding is specific to Japanese infertile women.

Patients make a decision to dispose of their embryos while coping with not just the loss of their embryo, but also their personal reproductive capability. Sublimation, letting go, and “Kuyo” can be regarded as their strategies for dealing with these simultaneous losses. These strategies, promoting spiritual and physical exercise, might have origins in Japanese culture emphasizes experiential transcendence in order to accept sadness such as loss and change in daily life [42]. Though religion was never mentioned influencing their decision, patients performed “Kuyo,” a commonly practiced Buddhist ceremony that has been absorbed and retained in Japan (now employed also in Shinto shrines). It is done from “thanks” and “apologies” towards hard worked things (from needles to brassieres), dead animals, and aborted fetuses (in this case, it is called mizuko) [43]. The concept of appreciation and apology overlapping is common in Japanese culture has been explained in cases of aborted fetuses as an expression of “apology” to a mizuko for having done wrong to it and merely expressing “thanks” to it for having vacated its place in the body of a woman and having moved on, leaving her relatively free of its physical presence [43]. Though, our participants said that “it is not equal to aborted fetus since it was intentionally made,” “Kuyo” eased them of not just from the guilt of parting with it in this life, but also from feeling thanks enabling them to feel not stigmatized as an infertile woman. Due to the nature of our interview being semi-structured, patients never mentioned religion; however, further research is required to conclude whether or not religion influences decision-making. It is essential for clinicians to be well prepared to support patients in their grief when expressions with similar meanings are used, realizing that they are trying to cope with the loss of both their embryo and their reproductive capability. The clinical implications of this model for medical professionals and counsellors are as summarized in Table 2.

**Table 2:** Clinical implications for medical professionals and counselors

	Clinical Implications
Step 2	· Offer sympathetic support by identifying the motives behind patients requesting information regarding their frozen embryo.· Facilitate discussion amongst family members and determine how much child and psychological supports are available.
Step 4	· Encourage couples to discuss the fate of their embryos from the time of its making.
Step 5	· Identify patient’s images toward research prior to offering information to obtain considerate and satisfactory consent for donation to research.· Recognize the possible alleviatory effect of research donation toward the guilt of disposal for many patients.
Common Theme	· Acknowledge that many patients have an identity of self as infertile and feel anxious that their reproductive capability is at stake during the decision making. · Support the grief work of patients by knowing that they are trying to cope with the loss of both the embryo and reproductive capability.

### Limitations

All participants were sampled from one institution. However, we collected a range of data from participants with various backgrounds and were able to reach a “theoretical saturation” [25]. Another limitation is that only 5 patients who had discarded their embryos agreed to be interviewed. This might be because of the lack of confidence in medical science as revealed in our results. In addition, many of the patients who choose to discard their embryos did so by not responding to our letter of notice. Our results might have been different if more patients in this group shared their experience, though gaining access to these patients is very difficult. We were unable to recruit participants less than 31 years of age to the interview. This is because patients tend to receive IVF treatment tends at a later age and even later when the letter of notice arrives. The result with younger patients may have been less pressured though we did not notice any difference between those in their 30’s and 40’s. We were only able to recruit a few participants without children for the interview process. The decision making conflicts might have been shown to be more intense if we had only interviewed patients without children, though reports indicate that there is no difference in the degree of difficulty in making the decision between those with a child or without [11]. More than half the participants were not religious and the others were mostly Buddhist. Therefore, this model might not be applicable for patients with different religious backgrounds. However, these findings will be useful for patients from these backgrounds in which such research has not thus for been undertaken.

## Conclusions

In conclusion, the 5 step decision-making model we describe in this paper with a major theme of “coming to terms with infertility,” makes an empirical contribution to understanding how patients decide the fate of their frozen embryos. We also found burden felt also by those continuing storage. The influence of Japanese moral/cultural values and beliefs were discovered to be central to this process, reflected in the how the embryos were conceptualized, as well as in the 3 strategies adopted to accept their infertility. A unique moral value empirically found in our study, “Mottainai,” gives us a new perspective on the philosophical arguments in medical ethics, mainly focusing on whether the embryo should be deemed a person (a child, early-life) or not, and sheds light on the actual issues surrounding infertile women who are faced to make a decision for the fate of their embryos. We have made suggestions for medical professionals and counselors to support not only the decision-making process, but also in assisting patients with their grief, perhaps by respecting patients’ pre-existing cultural beliefs. The results of this study contribute to the development of effective psychological support methods for patients who are uncertain how to decide the fate of their frozen embryos.

## Misc

Misao Fujita, Akihisa Fujimoto, Toshihiro Fujiwara, Tetsu Yano, Osamu Tsutsumi, Yuji Taketani and Akira Akabayashi contributed equally to this work

## Competing interests

The authors declare that there are no competing interests.

## Authors' contributions

ST and MF were involved in the construction of the study design, data collection and analysis, manuscript drafting and critical discussion. AF, TF, TY, OT and YT were involved in the data collection and critical discussion. AA was involved in the construction of the study design, data analysis, manuscript drafting and critical discussion. All authors read and approved the final manuscript.

## Pre-publication history

The pre-publication history for this paper can be accessed here:

http://www.biomedcentral.com/1472-6939/13/9/prepub
